# Cellular proteostasis: degradation of misfolded proteins by lysosomes

**DOI:** 10.1042/EBC20160005

**Published:** 2016-10-15

**Authors:** Matthew P. Jackson, Eric W. Hewitt

**Affiliations:** ^1^ School of Molecular and Cellular Biology and the Astbury Centre for Structural Molecular Biology, University of Leeds, Leeds LS2 9JT, U.K.

**Keywords:** amyloid, autophagy, chaperone-mediated autophagy, immunoglobulin light chain, lysosome, macroautophagy, mTOR, Parkinson's disease, protein aggregation, protein misfolding, proteostasis, rapamycin, TFEB, α-synuclein, β_2_-microglobulin

## Abstract

Proteostasis refers to the regulation of the cellular concentration, folding, interactions and localization of each of the proteins that comprise the proteome. One essential element of proteostasis is the disposal of misfolded proteins by the cellular pathways of protein degradation. Lysosomes are an important site for the degradation of misfolded proteins, which are trafficked to this organelle by the pathways of macroautophagy, chaperone-mediated autophagy and endocytosis. Conversely, amyloid diseases represent a failure in proteostasis, in which proteins misfold, forming amyloid deposits that are not degraded effectively by cells. Amyloid may then exacerbate this failure by disrupting autophagy and lysosomal proteolysis. However, targeting the pathways that regulate autophagy and the biogenesis of lysosomes may present approaches that can rescue cells from the deleterious effects of amyloidogenic proteins.

## Introduction

**Figure 1:**
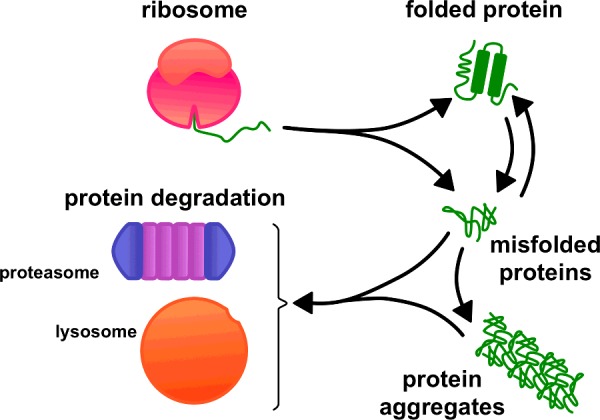
Cellular pathways of protein degradation remove misfolded proteins Proteins may either fail to fold after their synthesis or fold into their native structure and then subsequently misfold. Cells must dispose of these misfolded proteins and their aggregates in order to maintain proteostasis. Lysosomes and proteasomes represent the principal sites for the degradation of misfolded proteins and play complementary roles in proteostasis.

Proteostasis refers to the regulation of the cellular concentration, folding, interactions and localization of each the thousands of different proteins that comprise the proteome of an organism [1]. This is achieved by the close integration of cellular pathways responsible for the synthesis, folding, post-translational modification, trafficking and degradation of proteins. One vital function of this proteostatic network is the removal of any proteins that misfold. Conversely, the failure to degrade misfolded proteins can result in disease [2] (Figure 1).

## Cellular pathways for protein degradation

The principal sites for protein degradation in cells are lysosomes and proteasomes [3]. Both are involved in the constitutive turnover of cellular proteins; typically, short-lived proteins are degraded by proteasomes, whereas lysosomes are responsible for the degradation of long-lived proteins. Misfolded proteins also represent an important class of substrate for lysosomes and proteasomes [1]. These may include proteins that require degradation because they fail to fold after translation, such as those encoded by mutated sequences. Proteins that initially fold into their native conformations may also misfold due to environmental or stochastic factors and require degradation.

Proteasomes are multi-subunit protease complexes that are present in the cytoplasm of cells [3]. Most proteins degraded by proteasomes are tagged by polyubiquitination. These proteins are then fed into the core of the proteasome where they are cleaved by the internal protease activities into short peptides. Proteasomes can degrade a broad array of proteins, but they cannot cleave every protein, for example the extended polyglutamine tracts present in disease-associated mutant forms of huntingtin [4]. Moreover, proteins need to be unfolded before they can access the proteasome's catalytic core. Proteins and proteinaceous aggregates that resist unfolding by chaperone activities must be degraded elsewhere.

Lysosomes are membrane-bound cytoplasmic organelles that contain a plethora of different hydrolases, which degrade macromolecules and organelles [3]. These organelles have the capacity to degrade many fully folded, as well as misfolded and aggregated, proteins. Once within the lumen of the lysosome, proteins are hydrolysed by an array of proteases, known as cathepsins [5]. This generates free amino acids that can be used in cellular metabolism. Lysosomes therefore perform a complementary function to proteasomes in proteostasis, as shown by the lysosomal degradation of polyglutamine tracts [4].

## Sorting of misfolded proteins to the lysosome for degradation

**Figure 2:**
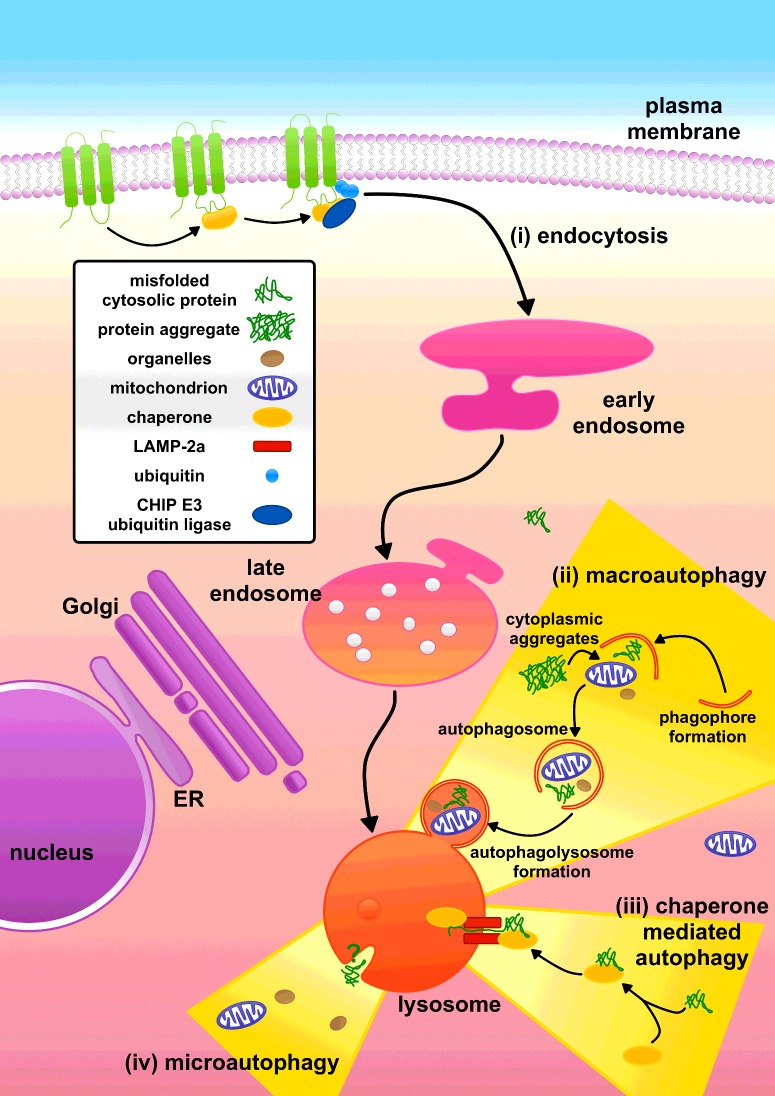
Multiple pathways deliver proteins to lysosomes for degradation (i) Misfolded plasma membrane proteins can be tagged for degradation via the E3 ubiquitin ligase CHIP. The resultant ubiquitinated proteins are endocytosed into early endosomes, which mature into late endosomes. The misfolded plasma membrane proteins are then delivered to lysosomes via the fusion of late endosomes with this organelle. (ii) Macroautophagy encapsulates cytoplasmic material, including soluble cytosolic proteins and protein aggregates, into double-membraned vesicles that then fuse with the lysosome. (iii) In CMA, the chaperone Hsc70 recognizes misfolded proteins and these are translocated into the lysosome by LAMP-2a. (iv) Microautophagy is associated with the formation of invaginations in the lysosomal membrane and could also contribute to the delivery of proteins into the lysosome. ER, endoplasmic reticulum. Adapted from [6,9].

The pivotal role of lysosomes in proteostasis is exemplified by the multiple pathways used by cells to traffic proteins to this organelle for degradation (Figure 2). Misfolded plasma membrane proteins are identified by CHIP [C-terminus of Hsc70 (heat-shock cognate 70)-interacting protein 70]. CHIP is responsible for recruitment of the E3 ubiquitin ligase, which ubiquitinates the plasma membrane protein, causing it to be endocytosed and ultimately delivered to the lysosome [6]. Cytoplasmic proteins are delivered to the lysosome by autophagy, of which there are three pathways: CMA (chaperone-mediated autophagy), macroautophagy and microautophagy [7]. However, whereas both CMA and macroautophagy play important roles in proteostasis, the role of microautophagy in the degradation of proteins is not well understood [8] and is therefore not discussed in this article. The endocytic pathway delivers extracellular proteins to lysosomes, as well as proteins that are resident at the plasma membrane and in endosomes [6].

### Delivery of misfolded proteins to lysosomes via macroautophagy

Macroautophagy encapsulates cytoplasmic material, including cytosolic macromolecules and even entire organelles, into characteristic double-membraned vesicles known as autophagosomes [6,10] (Figure 2). This cellular degradation pathway is constitutive, although macroautophagy can be enhanced by stresses such as starvation. The mTOR (mammalian target of rapamycin) complex regulates macroautophagy [10]. During the initial hours of nutrient-starved conditions or upon the addition of rapamycin, macroautophagy is activated. Macroautophagy initiation is controlled by ULK1 (Unc-51-like kinase 1), mTOR negatively regulates ULK1 by phosphorylation. ULK1 interacts with the class III PI3K (phosphoinositide 3-kinase) complex, which comprises Vps34 (vacuolar protein sorting 34), the adapter protein p150 and Beclin-1. Initiation consists of formation of an omegasome which elongates to form a phagophore. This phagophore elongates further, encapsulating its cargo, before sealing to produce an autophagosome. The autophagosome then fuses with a lysosome, delivering its contents for degradation by lysosomal hydrolases [10].

Macroautophagy enables folded proteins, as well as misfolded proteins and protein aggregates, to be delivered to the lysosome without needing to first unfold these proteins with chaperones [10]. Thus macroautophagy can complement the proteasome and CMA by degrading proteins and proteinaceous aggregates that resist unfolding by chaperone activities. Initially, macroautophagy was thought to be a non-selective mechanism for the turnover of proteins. Owing to its ability to encapsulate large volumes of cytoplasmic material, macroautophagy would enable both soluble proteins as well as proteinaceous aggregates to be delivered to the lysosome. However, more recent studies have shown that protein aggregates formed as a consequence of the failure of proteasomes to degrade proteins can be targeted selectively to the macroautophagy pathway. This involves recognition by specific adaptor proteins, such as p62, which link the aggregates to the assembling autophagosome [11].

### Delivery of misfolded proteins to lysosomes via CMA

**Figure 3:**
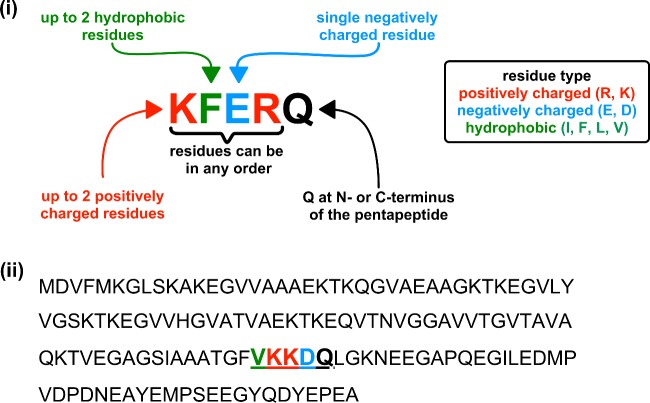
Proteins are recognized for CMA by a CMA-targeting motif (**i**) The CMA motif may contain up to two hydrophobic residues [isoleucine (I), phenylalanine (F), leucine (L) or valine (V)], up to two positive residues [arginine (R) or lysine (K)] and a single negatively charged residue [glutamate (E) or aspartate (D)] flanked at either the N- or C-terminus of the pentapeptide by a single glutamine (Q) residue. This is often referred to as a KFERQ or KFERQ-like motif [12]. (**ii**) Protein sequence of α-synuclein highlighting the CMA motif (bold and underlined) and potential CMA-targeting motif residues.

CMA involves the identification of misfolded proteins by Hsc70, a cytosolic chaperone [12] (Figure 2). Proteins degraded by CMA have a five-amino-acid motif with the consensus sequence KFERQ (Lys-Phe-Glu-Arg-Gln) (Figure 3). KFERQ-like motifs are present in approximately one-third of soluble cytosolic proteins. The KFERQ motif is buried in natively folded proteins. Misfolding of proteins may expose KFERQ-like motifs enabling Hsc70 binding. The substrate protein is then unfolded and translocated across the lysosome membrane. This process requires Hsc70 on both sides of the lysosomal membrane and the binding of the substrate protein to LAMP-2a (lysosome-associated membrane protein 2a) [12]. Binding drives the multimerization of LAMP-2a facilitating the translocation of the protein substrate across the lysosomal membrane [12].

### Delivery of misfolded proteins to lysosomes via the endocytic pathway

The cytosol is not the only cellular location in which proteins can misfold, and lysosomes are also involved in the degradation of misfolded plasma membrane proteins. Lysosomes are the terminus of the endocytic pathway, hence enabling plasma membrane proteins to be trafficked for degradation by lysosomal hydrolases. This is well documented for receptor down-regulation in cell signalling pathways, in which ligand-bound plasma membrane receptors are ubiquitinated by E3 ubiquitin ligases, with the ubiquitin tag acting as a signal for the endocytic trafficking of the receptor to the lysosome [6,9]. A similar process can be used to remove misfolded proteins from the plasma membrane. The E3 ubiquitin ligase CHIP, which recognizes misfolded proteins that are bound to chaperones, ubiquitinates the cytoplasmic domains of misfolded membrane proteins [6]. For example disease-associated mutations in the hERG (human *ether-a-go-go*-related gene) K^+^ channel result in its CHIP-dependent ubiquitination and trafficking via the endocytic pathway to lysosomes for degradation [13].

## Amyloid diseases and their interrelationship with protein degradation by lysosomes

Amyloid diseases, collectively known as the amyloidoses, represent a failure in proteostasis. This is because proteins are able to misfold into aggregates that are not cleared effectively by the pathways of protein degradation [14]. Irrespective of the precursor protein's sequence or structure, amyloid formation is associated with proteins aggregating into fibrils with a cross-β molecular architecture [15,16]. Deposits of amyloid can form within cells, such as in Parkinson's disease, although, in many diseases, amyloid forms extracellular deposits, e.g. DRA (dialysis-related amyloidosis) [15]. Amyloid fibrils, as well as oligomers formed during either the assembly or the disassembly of fibrils, can be toxic to cells by damaging cellular membranes, disrupting cellular physiology and may ultimately cause cell death [15,16]. Crucially, one important element of cellular physiology that can be disrupted by amyloid is protein degradation by lysosomes. This may not only exacerbate amyloid formation by increasing the level of proteins that can assemble into amyloid, but also have an impact on other cellular processes by reducing the degradation of other proteins.

The pathological interplay between lysosomes and amyloid is exemplified in Parkinson's disease [17]. In Parkinson's disease, α-synuclein aggregates into amyloid that is a major component of Lewy bodies which are cytoplasmic inclusions that are a primary histological hallmark of this neurodegenerative disorder [18]. α-synuclein amyloid can also be transmitted between cells, seeding aggregation of the protein in previously healthy cells [19]. Lysosomes are an important site for the degradation of α-synuclein [12], as such any reduction in either autophagy or in the capacity of lysosomes to degrade α-synuclein will result in increased levels of this protein, a major risk factor in its aggregation. Reduced proteolytic activity in lysosomes can also enhance the transmission of α-synuclein amyloid between cells [20]. A reduced capacity of cells to degrade proteins is a feature of aging and may explain, at least in part, the increased incidence of sporadic Parkinson's disease with age [1]. Moreover, cathepsin D, LAMP-2a and Hsc70 are reduced in dopaminergic neurons of subjects with Parkinson's disease, suggesting an impairment in the ability of neurons to deliver α-synuclein to lysosomes and in the subsequent degradation of the protein [21].

CMA is thought to be a major mechanism for the degradation of α-synuclein in healthy cells [12], with α-synuclein containing the KFERQ-like motif FVKKDQ (Phe-Val-Lys-Lys-Asp-Gln) (Figure 3). The A30P and A53T dominant-negative mutants of α-synuclein, which are associated with early onset familial forms of Parkinson's disease, inhibit CMA [22]. These mutant proteins bind to LAMP-2a, but are not transported across the lysosome membrane, resulting in the inhibition of CMA [22]. Similarly, high levels of wild-type α-synuclein can disrupt CMA [23]. Crucially, inhibition of CMA not only promotes the formation of α-synuclein amyloid, presumably due to increased levels of the protein, but also affects the degradation of other cellular proteins. One such protein is the neuronal survival factor MEF2D (myocyte enhancer factor 2D), which, as a result of reduced CMA, accumulates in an inactive form in the cytoplasm [23]. In addition, α-synuclein amyloid can disrupt macroautophagy, as Lewy body-like α-synuclein amyloid formed in cultured cells, resist degradation and cause a reduction in the clearance of autophagosomes [24].

Although Parkinson's disease is associated with formation of amyloid fibrils within the cytoplasm of cells, in many other amyloid disorders fibrils form extracellular deposits [15]. Lysosomes, however, are also vulnerable to extracellular amyloids, as material from outside of cells can access lysosomes via the endocytic pathway. We have shown, using β_2_m (β_2_-microglobulin), the culprit protein of DRA, that, if the amyloid fibrils are small enough, ∼300 nm in length, they can be readily endocytosed by cells and trafficked to lysosomes [25]. Once within lysosomes, β_2_m fibrils resist degradation and inhibit the proteolysis of endocytosed proteins by lysosomes [25]. Whether this inhibition is due to overwhelming lysosomal proteases with hard-to-digest protein or another mechanism is unclear, but the inhibition of protein degradation in lysosomes could also have an impact on autophagy. A role for lysosome dysfunction has also been reported in immunoglobulin light chain amyloidosis [26]. Exposure of cells to immunoglobulin light chain amyloid results in lysosome dysfunction and the disruption of autophagy, specifically reducing the clearance of autophagosomes [26]. This has parallels with the effects of intracellular α-synuclein aggregates on macroautophagy [24].

## Enhancing protein degradation by lysosomes to reduce amyloid toxicity

Since many studies suggest an interrelationship between amyloid diseases and dysfunction of protein degradation in lysosomes, could enhancing autophagy and lysosome function provide a way to treat these devastating disorders? mTOR is a negative regulator of macroautophagy and its inhibition with rapamycin stimulates macroautophagy [10]. Rapamycin can also restore the clearance of autophagosomes in cells exposed to light chain amyloid and reduce toxicity associated with this amyloid [26]. Overexpression of sirtuin-1 can also suppress the formation of α-synuclein amyloid in cell and animal models and it is thought to act via the induction of autophagy [27]. An alternative target is TFEB (transcription factor EB), which functions as a master regulator of lysosomal biogenesis and autophagy [28]. Overexpression of TFEB or its activation with HPβCD (2-hydroxypropyl-β-cyclodextrin) promotes the clearance of α-synuclein amyloid via the enhancement of macroautophagy in cells overexpressing α-synuclein [28]. Moreover, TFEB overexpression in a mouse model of Parkinson's disease enhances expression of proteins associated with macroautophagy and lysosomes, and crucially protects neurons from α-synuclein toxicity [29].

Activating lysosomal biogenesis and autophagy may be one approach to enhance lysosome function in amyloid diseases, but another is to target the fibrils themselves. Hsp70 (heat-shock protein 70) is a chaperone whose expression is induced by cellular stress, and, although predominantly cytosolic, the protein is also localized to the lysosome. We have found that incubation of cells with Hsp70 can prevent the inhibition of lysosomal proteolysis by β_2_m fibrils [30]. Hsp70 may act by binding to and stabilizing β_2_m fibrils preventing their disassembly into toxic oligomers at the mildly acidic pH they would encounter in the endocytic pathway [30].

## Conclusions

Lysosomes are a key component of the cellular machinery that maintains proteostasis, and these organelles play a central role in the degradation of misfolded proteins and their aggregates. The importance of lysosomes in proteostasis is highlighted by the multiple cellular pathways used by cells to identify misfolded proteins for delivery to lysosomes. CMA, macroautophagy and the endocytic pathway traffic misfolded proteins to lysosomes. The role of microautophagy is less well understood, but, as a pathway for delivery of material to lysosomes, it could also contribute to proteostasis. Amyloid diseases in effect correspond to a failure in proteostasis. In some instances, they may be associated with a reduced ability of cells to deliver proteins to lysosomes, thus resulting in the accumulation of proteins that can assemble into amyloid. Aging is associated with a reduced cellular proteolytic capacity and may, at least in part, explain why amyloid diseases are more common in older subjects. Amyloid precursors and amyloid can also disrupt the delivery of proteins to the lysosome via autophagy and impair proteolysis in lysosomes. Are amyloid diseases therefore a vicious cycle in which amyloid exacerbates the reduction in cellular proteolytic capacity, resulting in yet further production of amyloid? Whereas amyloid can exhibit toxic properties, disruption of autophagy will also have an impact on other cellular functions and may be a downstream pathological consequence of amyloid formation. Disruption of lysosomal proteolysis could also have an impact on the degradation of plasma membrane proteins. Despite the increasing evidence of impaired lysosome function in amyloid disease, lysosomes are an underutilized drug target in the treatment of these disorders. Increasing autophagy and the biogenesis of lysosomes via targeting TFEB-dependent transcription or mTOR signalling may provide new therapeutic routes to treat these devastating disorders.

## Summary

- The degradation of misfolded proteins is an essential element of proteostasis.
- Lysosomes are degradative organelles that are responsible for the breakdown of proteins and other cellular components.
- Misfolded proteins are sorted to lysosomes via chaperone-mediated autophagy, macroautophagy and endocytosis.
- Amyloid diseases, such as Parkinson's disease, represent a failure in proteostasis in which proteins misfold into amyloid aggregates.
- Amyloid disrupts autophagy and the degradation of proteins in lysosomes, thus exacerbating this failure in proteostasis.
- Promoting autophagy and the formation of new lysosomes may protect against amyloid diseases.

## Abbreviations:

β_2_m: β_2_-microglobulin
CHIP: C-terminus of Hsc70-interacting protein 70
CMA: chaperone-mediated autophagy
DRA: dialysis-related amyloidosis
Hsc70: heat-shock cognate 70
Hsp70: heat-shock protein 70
LAMP-2a: lysosome-associated membrane protein 2a
mTOR: mammalian target of rapamycin
TFEB: transcription factor EB
ULK1: Unc-51-like kinase 1
